# Metabolomic profiling identifies novel associations with Electrolyte and Acid-Base Homeostatic patterns

**DOI:** 10.1038/s41598-019-51492-3

**Published:** 2019-10-21

**Authors:** Cristina Menni, Linsay McCallum, Maik Pietzner, Jonas Zierer, Alisha Aman, Karsten Suhre, Robert P. Mohney, Massimo Mangino, Nele Friedrich, Tim D. Spector, Sandosh Padmanabhan

**Affiliations:** 1 Department of Twin Research & Genetic Epidemiology, King’s College London, London, UK; 2 Institute of Cardiovascular and Medical Sciences, College of Medical, Veterinary and Life Sciences, University of Glasgow, Glasgow, UK; 3 Institute of Clinical Chemistry and Laboratory Medicine, University Medicine Greifswald, Greifswald, Germany; 4 DZHK (German Center for Cardiovascular Research), partner site Greifswald, Greifswald, Germany; 5 Department of Physiology and Biophysics, Weill Cornell Medical College in Qatar, Education City, Qatar Foundation, Doha, Qatar; 6 Metabolon, Inc., Durham, USA

**Keywords:** Biomarkers, Nephrology

## Abstract

Electrolytes have a crucial role in maintaining health and their serum levels are homeostatically maintained within a narrow range by multiple pathways involving the kidneys. Here we use metabolomics profiling (592 fasting serum metabolites) to identify molecular markers and pathways associated with serum electrolyte levels in two independent population-based cohorts. We included 1523 adults from TwinsUK not on blood pressure-lowering therapy and without renal impairment to look for metabolites associated with chloride, sodium, potassium and bicarbonate by running linear mixed models adjusting for covariates and multiple comparisons. For each electrolyte, we further performed pathway enrichment analysis (PAGE algorithm). Results were replicated in an independent cohort. Chloride, potassium, bicarbonate and sodium associated with 10, 58, 36 and 17 metabolites respectively (each P < 2.1 × 10^−5^), mainly lipids. Of all the electrolytes, serum potassium showed the most significant associations with individual fatty acid metabolites and specific enrichment of fatty acid pathways. In contrast, serum sodium and bicarbonate showed associations predominantly with amino-acid related species. In the first study to examine systematically associations between serum electrolytes and small circulating molecules, we identified novel metabolites and metabolic pathways associated with serum electrolyte levels. The role of these metabolic pathways on electrolyte homeostasis merits further studies.

## Introduction

Electrolytes have a crucial role in maintaining health and their serum levels are homeostatically maintained within a narrow range by multiple mechanisms usually involving the kidneys^1^. Sodium is the principal cation and osmotic agent in the extracellular fluid (ECF) compartment, while potassium is the main cation in the intracellular fluid (ICF) compartment with the two compartments separated by the cell membrane. The ECF volume is essentially preserved by the factors controlling the body sodium content mainly by the kidneys^1^. Of the total body sodium, 44% is in the ECF, 9% in the ICF and the remaining 47% in bone^2^. Only about half of the bone sodium is exchangeable and the rest are osmotically inactive. The ICF contains almost 98% of potassium^1,3^ and 75% of the body potassium stores is in skeletal muscle^1^. The sodium-potassium ATPase pump is the most important determinant of potassium distribution and the activity of the pump itself is increased by catecholamines and insulin^4–7^. The major anions balancing the cations are chloride and bicarbonate in the ECF while it is proteins and phosphates in the ICF. Regulation of the internal distribution of potassium is efficiently maintained by the kidneys as the movement of as little as 2% of the ICF potassium to the ECF can result in a potentially fatal increase in the serum potassium concentration^1^. The homeostatic maintenance of the controlled partition of fluid and electrolytes in different body compartments is essential for health, and in clinical medicine, most measurements of electrolyte concentration are performed on the extracellular fluid compartment, notably the blood serum. At a cellular level, metabolic processes are crucial for the regulation of fluid and electrolytes, the maintenance of serum protein level, and control of the amounts of sugar (glucose) and fats (lipids) in the blood the *milieu interieur*^8^.

Understanding the relationship between serum electrolytes and small molecules in circulation may generate insights into homeostatic mechanisms that are orthogonal to kidney functional status as proteins, phospholipids, cholesterol, and neutral fats account for 90% of the mass of dissolved solutes in the serum^9^. The aim of this study is to use metabolomics profiling to discover molecular markers and pathways associated with serum electrolyte levels.

## Results

A total of 1523 adults from the TwinsUK cohort not on BP-lowering therapy and without renal impairment were included in the analysis of 592 fasting serum metabolites. The demographic characteristics of the study population are presented in Table 1. Chloride, potassium, bicarbonate, and sodium were found to correlate with 10, 58, 36 and 17 metabolites respectively (each P < 2.11 × 10^−5^ after multivariate adjustment; Table S1 in the Supplementary Material).

**Table 1:** Demographic characteristics of the study population.

Variable	TwinsUK	SHIP
N	1523	938
Females n(%)	1462 (95.99%)	522 (55.6%)
Age, yrs	50.29 (8.20)	49.5(10.75)
Bicarbonate, mmol/L	24.99 (2.65)	NA
BMI, kg/m²	25.13 (4.11)	27.2 (4.5)
Chloride, mmol/L	104.75 (2.49)	NA
Creatinine, mmol/L	73.94 (9.54)	73.6 (13.7)
DBP, mmHg	77.08 (10.33)	76.7 (9.8)
eGFR, mL/min/1.73 m^2^	79.26 (11.89)	93.5 (15.4)
Potassium, mmol/L	4.19 (0.33)	4.49 (0.35)
Sodium, mmol/L	140.99 (2.08)	139.3 (2.2)
SBP, mmHg	120.38 (14.60)	124.0 (16.7)
Triglycerides, mmol/L	1.16 (0.70)	NA

Note: Characteristics are expressed in mean (SD) for all variables, except gender (%).

### Metabolites-electrolytes associations

Backward linear regressions identified 6 metabolites independently associated with sodium (R^2^ = 0.07), 4 with chloride (R^2^ = 0.08), 8 with potassium (R^2^ = 0.16), and 13 with bicarbonate (R^2^ = 0.27), (Table 2). Serum sodium and chloride levels show significant direct association predominantly with amino-acid metabolites. In the methionine-cysteine pathway, serum sodium is significantly associated with cystine (Beta(SE) = 0.31(0.07), P = 6.78 × 10^−6^) and cystathionine (0.39(0.06), P = 2.67 × 10^−10^) and serum chloride with N-acetyl methionine (0.33(0.07), P = 7.65 × 10^−7^) (Table S1). In the urea cycle pathway, serum sodium directly associated with pro-hydroxy-pro (0.29(0.05), P = 1.00 × 10^−7^) and N-acetylcitrulline (0.31(0.06), P = 3.01 × 10^−8^). N-acetylputrescine (polyamine pathway) is inversely associated with serum sodium (−0.23(0.05), P = 5.61 × 10^−6^), while aspartate is inversely associated with serum chloride (−0.40(0.09), P = 7.34 × 10^−6^). Other significant associations are DHEA-S for serum sodium (0.25(0.06), P = 1.61 × 10^−5^) and the inverse association of γ-glutamyl-ε-lysine (−0.30(0.07), P = 9.11 × 10^−6^) and oxalate (−0.42(0.08), P = 1.37 × 10^−7^) for serum chloride (Table S1).

**Table 2:** List of metabolites independently associated with serum potassium or sodium levels in TwinsUK and replicated in SHIP, when applicable*.

METABOLITE	TwinsUK			SHIP		
	Beta	SE	P	Beta	SE	P
***SERUM POTASSIUM***						
sphingomyelin (d18:1/20:0, d16:1/22:0)*	0.04	0.01	1.20 × 10^−6^			
dihydroorotate	−0.05	0.01	1.56 × 10^−7^			
indolelactate	0.04	0.01	9.47 × 10^−7^	0.3	0.09	1.33 × 10^−3^
fumarate	−0.07	0.01	7.24 × 10^−16^			
myristoleate (14:1n5)	−0.06	0.01	1.97 × 10^−14^	−0.43	0.08	3.17 × 10^−7^
2-isopropylmalate	−0.05	0.01	2.53 × 10^−8^			
10-heptadecenoate (17:1n7)	−0.05	0.01	1.07 × 10^−8^	−0.28	0.09	1.01 × 10^−3^
linolenate [alpha or gamma; (18:3n3 or 6)]	−0.05	0.01	1.94 × 10^−12^	−0.19	0.09	4.68 × 10^−2^
***SERUM SODIUM***						
cystine	0.31	0.07	6.78 × 10^−6^			
pro-hydroxy-pro	0.29	0.05	1.00 × 10^−7^	0.04	0.01	4.76 × 10^−3^
cystathionine	0.39	0.06	2.67E-10			
N-acetylputrescine	−0.23	0.05	5.61 × 10^−6^			
N-acetylcitrulline	0.31	0.06	3.01 × 10^−8^			
dehydroisoandrosterone sulfate (DHEA-S)	0.25	0.06	1.61 × 10^−5^	0.01	0.01	5.12 × 10^−1^
***SERUM BICARBONATE***						
suberate (octanedioate)	0.28	0.05	1.84 × 10^−8^			
N-acetylphenylalanine	0.45	0.06	2.15 × 10^−15^			
glycerophosphorylcholine (GPC)	0.25	0.06	1.50 × 10^−5^			
lysine	−0.33	0.06	7.35 × 10^−8^			
glycerophosphoinositol*	0.43	0.06	4.31 × 10^−13^			
threonine	−0.34	0.06	3.92 × 10^−8^			
4-hydroxychlorothalonil	0.34	0.06	2.43 × 10^−9^			
1-docosapentaenoyl-GPC (22:5n3)*	0.26	0.06	1.04 × 10^−5^			
methyl indole-3-acetate	0.27	0.06	1.64 × 10^−6^			
phenylpyruvate	−0.30	0.06	1.31 × 10^−7^			
citrulline	0.44	0.06	2.00 × 10^−14^			
prolylproline	−0.30	0.06	3.20 × 10^−6^			
gamma-glutamylthreonine*	−0.40	0.06	7.05 × 10^−10^			
***SERUM CHLORIDE***						
aspartate	−0.40	0.09	7.34 × 10^−6^			
N-acetylmethionine	0.33	0.07	7.65 × 10^−7^			
oxalate (ethanedioate)	−0.42	0.08	1.37 × 10^−7^			
gamma-glutamyl-epsilon-lysine	−0.30	0.07	9.11 × 10^−6^			

^*^SHIP metabolomics was measured by Metabolon Inc, using an older version of the platform employed for TwinsUK that covers less metabolites. Replication was performed only for metabolites measured in the two cohorts.

Serum potassium exhibited significant associations with tricarboxylic acid (TCA) cycle intermediates and metabolites involved in lipid metabolism pathways, including medium-chain fatty acids, long-chain and polyunsaturated fatty acids, dicarboxylic acids, acylcarnitines, and glycerol. Each of these metabolites were inversely correlated with serum potassium; only sphingomyelin showed a direct correlation. Associations between non-lipid-related metabolites and serum potassium were also observed (Table S1).

Serum bicarbonate concentrations significantly associated with amino-acid as well as lipid species. Most of the amino-acids were positively associated, including suberate, N-acetylphenylalanine, glycerophosphorylcholine (GPC), and citrulline, while lysine, threonine, and phenylpyruvate were inversely associated. All the lipids, 4-hydroxychlorothalonil, methyl indole-3-acetate, and 1-docosapentaenoyl-GPC (22:5n3) were positively associated.

### Metabolite association by physicochemical patterns

**Figure 1:**
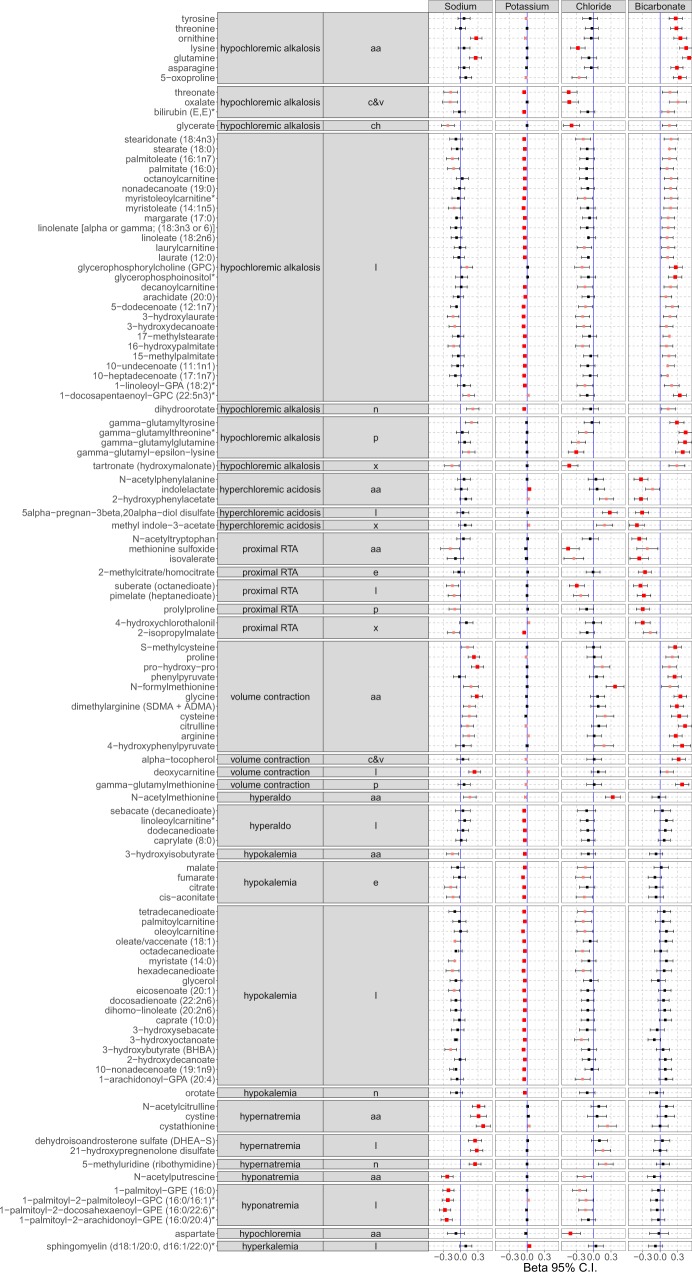
Metabolite associations by electrolyte and acid-base physicochemical patterns. aa = amino-acids, c&v = cofactors & vitamins, ch = carbohydrates, l = lipids, n = nucleotides, p = peptides, x = xenobiotics.

The results of metabolite association by physicochemical patterns are presented in Fig. 1. Hypochloremic alkalosis pattern is evident with metabolites both in the amino-acid and lipid groups, while volume contraction alkalosis pattern is predominantly seen with amino-acids and only one lipid (deoxycarnitine) metabolite. The amino-acid associations for hypochloremic alkalosis show highly significant associations with serum bicarbonate while the associations with potassium were only minor. In contrast, the lipid associations showed nominally significant bicarbonate and highly significant potassium associations. Nearly all the amino-acids in the volume contraction alkalosis group showed significant sodium and bicarbonate associations with borderline to no potassium associations. Aspartate was solely associated with hypochloremia with no associations with bicarbonate or other electrolytes. Interestingly, lipid metabolites were consistently significantly associated with hypokalemia either as a solo association or as part of a physicochemical pathway, while other metabolites showed variable association with potassium as part of physicochemical pathways. This contrasts with sodium or bicarbonate associations which showed the significant metabolites distributed across amino-acids and lipids.

### Replication

We replicated the metabolites independently associated with either serum potassium or serum sodium in the SHIP-TREND cohort. Out of the 8 metabolites independently associated with serum potassium levels, 4 (indolelactate, myristoleate, 10-heptadecenoate, and linolenate [alpha or gamma]) were also measured in SHIP-TREND and were significantly replicated (Table 2), while out of the 6 metabolites independently associated to serum sodium, two (pro-hydroxy-pro and DHEAS) were also present in SHIP-TREND and pro-hydroxy-pro was significantly replicated.

**Figure 2:**
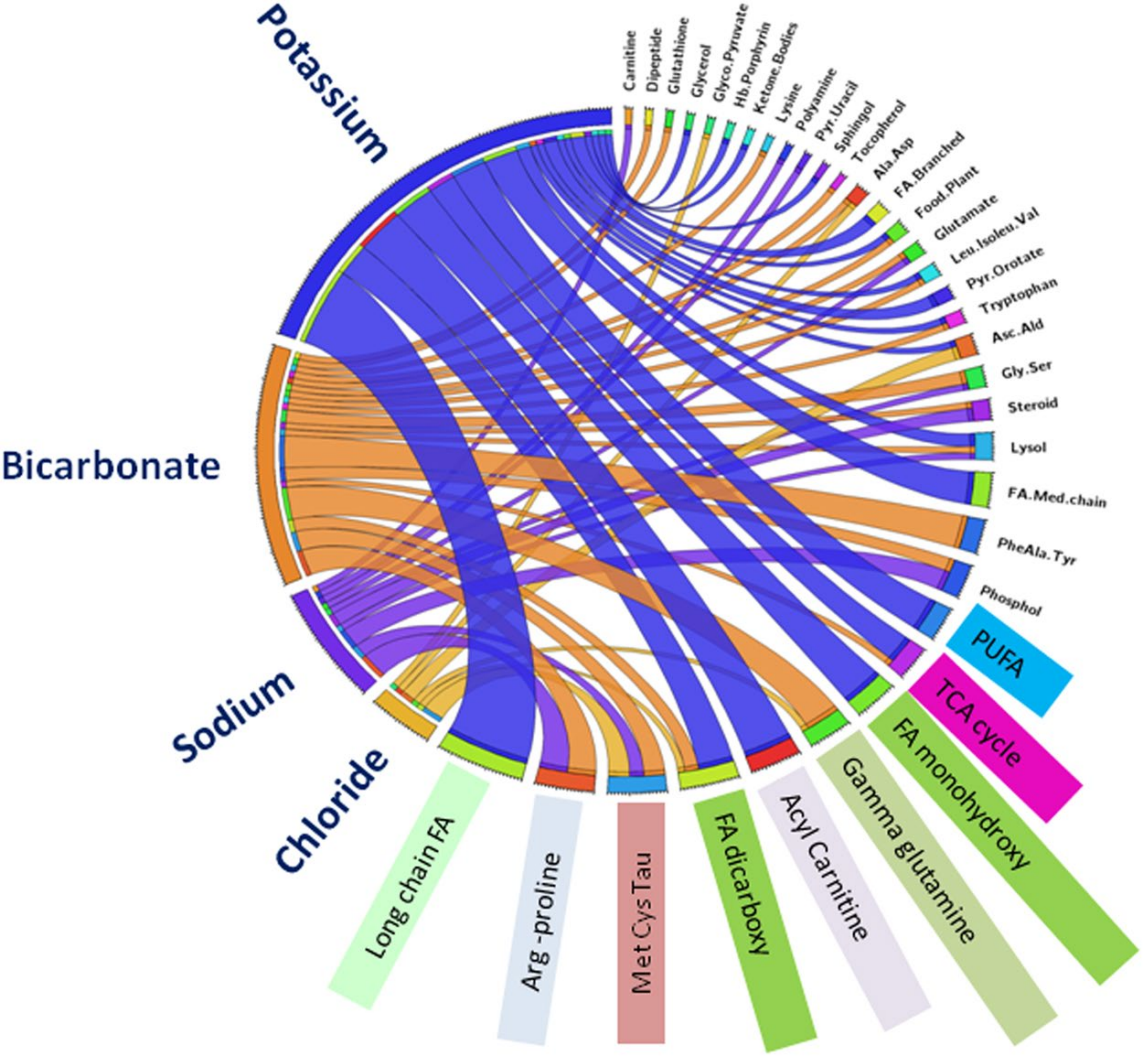
Pathway analysis based on the metabolite associations with electrolytes in TwinsUK. All pathways enriched for potassium are colour coded in blue, for bicarbonate in orange, for sodium in purple and for chloride in yellow.

Pathway enrichment analyses (Fig. 2) show long chain fatty acid, PUFA, monohydroxy fatty acid and acylcarnitine pathways specifically associated with just serum potassium. Dicarboxylic fatty acid pathway is enriched for potassium, chloride and bicarbonate while the TCA cycle pathway is associated with serum potassium and serum bicarbonate. Arginine-proline pathway is associated with only sodium and chloride.

## Discussion

This is the first study to examine systematically associations between serum electrolytes and small circulating molecules. We identified yet unknown dependencies between metabolites and metabolic pathways associating with serum electrolyte levels, and replicated some of the top associations in an independent cohort. Of all the electrolytes, serum potassium showed the most significant associations with individual fatty acid metabolites and specific enrichment in fatty acid pathways. In contrast, serum sodium and bicarbonate showed associations predominantly with amino-acids. Mapping electrolyte and bicarbonate patterns based on the Stewart physicochemical model of acid-base chemistry^10,11^, we identify novel correlations between amino-acid and lipid metabolite associations with homeostatic patterns and further refine the electrolyte associations that are truly independent. These associations may be the consequences or the causes of these physicochemical patterns and they warrant replication and further research.

The major finding from this work is an inverse association between serum potassium and a range of long-chain and medium-chain fatty acids. A previous study of male Wistar rats showed induced potassium deficiency was associated with decreased levels of total lipids, phospholipids, and glycerol and increased levels of free fatty acids, implying that potassium-deficient rats use fatty acids as a substrate for oxidative metabolism^12^. Consistently, our findings indicate that low potassium is associated with increased fatty acids, glycerol, ketoacids, acylcarnitines and TCA cycle intermediates supporting an independent effect of serum potassium on lipolysis and fatty acid production. Furthermore, an increase in free fatty acids (FFA) is known to be a risk factor for insulin resistance and diabetes^13^. Two observational studies that have examined the association between serum potassium and risk of diabetes independent of antihypertensive use showed increased risk of type 2 diabetes with low potassium levels^14,15^. Our findings link low potassium with an increase in fatty acid suggesting a possible mechanism for increased risk of type 2 diabetes in relation to serum potassium, although this needs to be validated.

Serum potassium showed a direct association with sphingomyelin levels. A similar relationship was seen with serum sodium, but it did not attain statistical significance. In humans, sphingomyelins comprise nearly 85% of all sphingolipids and 10–20 mol% of the total serum membrane lipids^16^. Interestingly, the sphingolipids, sphingomyelin and ceramide have been shown to mediate loss of insulin sensitivity, to promote the characteristic diabetic proinflammatory state, and to induce cell death and dysfunction in important organs such as the pancreas and heart^17^.

Serum potassium also showed a significant inverse association with dihydroorotate (DHO) levels. Dihydroorotate dehydrogenase (DHODs) are flavin mononucleotide-containing enzymes that convert dihydroorotate to orotate in the only redox step in the de novo synthesis of pyrimidines^18^. The arthritis treatment drug leflunomide is a potent inhibitor of DHOD and a decrease in potassium levels is a known side-effect of this drug^19^. This indicates a possible role of DHOD in the regulation of serum potassium levels.

Here, serum sodium exhibited a predominantly significant direct association with several amino-acids and derivatives. The relationship between serum sodium and amino-acids may be related to the significant role played by the kidney in maintaining serum amino-acid concentrations; specifically, nearly 99% of all amino-acids filtered by the kidneys are reabsorbed in the proximal renal tubules and returned to serum^20,21^. The Na^+^ gradient is established across the cell by Na^+^-K^+^ ATPase, and inhibition of this pump by ouabain diminishes amino-acid uptake^22^. Among the amino-acids and related molecules, the most significant direct associations were with cysteine and its immediate upstream precursor cystathionine, which are components of the methionine-cysteine-taurine pathway. Our data show a direct relationship between circulating levels of cysteine and cystathionine and the electrolyte sodium. Cysteine may contribute to blood pressure control through multiple mechanisms including antioxidant effects that protect nitric oxide from the oxidative effects of reactive oxygen species (ROS), through modulation of the renin-angiotensin system^23^. Thus, the direct association between blood sodium and cysteine (and cystathionine) may reflect a compensatory mechanism by the body to modulate sodium-induced hypertension. Additionally, we also see two metabolites (21-hydroxypregnenolone and DHEA-S) from the adrenal-steroid synthesis pathway to feature within the top 20 associations with serum sodium, though given this should be taken with caution because of the sex imbalance of our discovery cohort.

The renal system provides a powerful mechanism to maintain acid-base balance within the body through modulation of bicarbonate levels in the blood, which help to keep the pH of the blood in the narrow range essential for life. Chloride is transported actively and passively with either HCO3^−^ or sodium. It is absorbed in both the small and large intestine^1^. There is a normal Cl^−^–HCO3^−^ exchange in the small bowel^1^. Acid-base balance and amino-acid metabolism are intimately related. Changes in acid-base balance influence the metabolic fate of many amino-acids^24^. Serum lysine shows significant inverse association with serum chloride and a direct association with serum bicarbonate. Administration of lysine monochloride is known to profoundly inhibit bicarbonate reabsorption and cause severe bicarbonate diuresis while lysine dichloride does not affect bicarbonate transport^25^. Citrulline malate has been shown to increase renal reabsorption of bicarbonate and we see a significant direct association between citrulline and bicarbonate^26^. Glutamine is the primary amino-acid involved in renal ammonia-genesis, a process intimately related to acid excretion^24^. The basic (cationic) amino-acids (lysine, arginine and histidine) yield neutral end-products plus a proton; sulfur (methionine and cysteine) amino-acids are also acidogenic because they generate sulfuric acid when oxidized^2^. The dicarboxylic (anionic) amino-acids (aspartate and glutamate, but not asparagine and glutamine) consume acid when oxidized and thus reduce the acid load of the diet^2^.

In this study, direct associations between serum bicarbonate and numerous protein amino-acids were observed, the strongest of which (Beta = 0.50) involved glutamine, which has the highest concentration in blood of all amino-acids. However, the bicarbonate associations all occurred as part of physicochemical patterns and we did not observe any bicarbonate association unaccompanied by association with other electrolytes.

We also note some study limitations. Our discovery sample consisted of females only. We have only replicated a fraction of the associations and we have not validated the physicochemical pathway signals. The metabolic associations observed do not necessarily have to be related to the renal system, but could also be related to metabolic alterations in the pancreas, liver, muscle, fat or other metabolically active organs. Further studies integrating urinary data will help dissect the renal contributions from others. Moreover, our association data provides only a snapshot on whether levels of proteins, phospholipids, cholesterol, and neutral fats have an impact on free circulating electrolyte concentrations and lack a mechanistic component. Notwithstanding, we identify metabolite pathways associated with complex homeostatic mechanisms which maintain electroneutrality and acid-base balance. This brings a common understanding of the physiological implications of shifts in electrolytes even among healthy individuals and may inform or support future physiological studies.

## Methods

### Study cohorts

#### Discovery cohort

Study subjects were twins enrolled in the TwinsUK registry, a national register of adult twins recruited as volunteers without selecting for any particular disease or trait^27^. All recruited twins were of the same sex. Here we analysed data from 1523 individuals, mainly females (96%) with a wide age range (32.8–74.7 years) without renal impairment (estimated glomerular filtration rate (eGFR) >60 mL/min per 1.73 m^2^) and not on any blood pressure (BP)-lowering medications. All individuals included in the analysis had metabolomics profiling and electrolytes measured in serum at the same time point. Serum sodium, potassium, chloride and bicarbonate were measured at St Thomas Hospital using the Kodak Ektachem dry chemistry analysers (Johnson and Johnson Vitros Ektachem machine which uses thin-film’dry’ chemistry technology to perform colorimetric and potentiometric analyses. Hospital laboratories in the UK are subject to robust external quality control schemes (NEQAS [National External Quality Assessment Service]). Direct potentiometric electrolyte assays were performed using ion-selective electrodes which has a selective membrane in contact with both the test solution (patient’s sample) and an internal filling solution (containing the test ion at a fixed concentration). The coefficients of variation for chloride, bicarbonate, sodium and potassium were 1%, 1.5%, 0.9% and 4.8% respectively.

The study was approved by St. Thomas’ Hospital Research Ethics Committee. All participants provided informed written consent. The investigation conforms to the principles outlined in the Declaration of Helsinki. TwinsUK metabolomics, expression and phenotypic data are publicly available upon request on the department website (http://www.twinsuk.ac.uk/data-access/accessmanagement/).

#### Replication cohort

The replication cohort consisted of individuals not on BP-lowering therapy and without renal impairment drawn from the Study of Health in Pomerania (SHIP-TREND), a population-based research project in West Pomerania, a rural region in north-east Germany^28^. In total, 938 individuals (aged 20–79 years) with fasting serum metabolomic profiles available using the Metabolon, Inc. (Durham, USA) platform (for details see^29^) and with measure potassium and sodium were analysed.

### Metabolomic profiling

Non-targeted metabolite detection and quantification was conducted by the metabolomics provider Metabolon, Inc. (Durham, USA)on fasting serum samples, as described previously^30^. The metabolomic dataset measured by Metabolon includes 592 known metabolites containing the following broad categories - amino-acids, peptides, carbohydrates, energy intermediates, lipids, nucleotides, cofactors and vitamins, and xenobiotics.

### Statistical analysis

Statistical analysis was carried out using Stata version 11 and R. Quality control of metabolomics data was carried out as previously described^30^. We inverse normalised the data as the metabolite concentrations were not normally distributed. To avoid spurious false-positive associations due to small sample size, we excluded metabolic traits with more than 20% missing values leaving for analysis 592 metabolites of known chemical identity. We imputed the missing values using the minimum run day measures.

We looked for metabolites (outcome) associated with chloride, sodium, potassium and bicarbonate (exposure) by running linear mixed models adjusting for age, body mass index (BMI), gender and family relatedness. We corrected for multiple comparisons using Bonferroni correction, thus giving a significant threshold of *P* = 2.1 × 10^−5^ (0.05/(592 metabolites x 4 phenotypes). We then used a stepwise backward regression model to identify a set of metabolites that were significantly associated with each phenotype using P < 0.01 as cut-off threshold.

We replicated the metabolites independently associated with serum sodium or serum potassium in 938 individuals from SHIP using linear regressions and adjusting for age, BMI and gender. Serum chloride and serum bicarbonate measurements were not available for the SHIP-TREND cohort.

Finally, for each electrolyte, we performed pathway enrichment analysis in the TwinsUK data using the PAGE algorithm implemented in the R-package piano^31^. Enrichment tests were performed for each given pathway while taking into account the sign of the associations. Statistical significances of all enrichment analyses were assessed using empirical p-values estimated from a background distribution of 10,000 random permutations of the variable labels, and multiple testing correction was used to correct for the number of tested pathways.

### Physicochemical pathways

Acid–base balance and electrolyte homeostasis are intricately connected to maintain the internal

environment of the body within narrow and rigidly controlled limits. Thus, the study of metabolomic associations of electrolytes need to be considered in the context of these homeostatic processes. We mapped common electrolyte and acid-base patterns, based on the bicarbonate-centric physicochemical model and electroneutrality, to metabolite associations. We classified metabolites showing a hypochloremic alkalosis pattern, if they showed a nominally significant positive association with serum bicarbonate and a negative association with serum chloride and serum potassium. We contrast this from volume contraction alkalosis, which we defined as metabolites showing nominally significant positive association with serum bicarbonate, positive association with serum sodium and variable association with serum potassium and chloride. Hyperchloremic acidosis and proximal renal tubular acidosis patterns were defined by nominally significant negative association with serum bicarbonate along with positive association with serum chloride in the former and negative association with serum chloride in the latter. Pure electrolyte associations were defined as hypo- or hyper- natremia or kalemia when the metabolites showed null association with serum bicarbonate.

## Supplementary information

Supplementary Material
