# Supplementary material for: Metabolomic profiling identifies novel associations with Electrolyte and Acid-Base Homeostatic patterns
Source: Sci Rep. 2019 Oct 21;9:15088. doi: 10.1038/s41598-019-51492-3 (PMC6803625; doi:10.1038/s41598-019-51492-3)
Supplement: Supplementary file 1 — Supplementary Material [file 41598_2019_51492_MOESM1_ESM.pdf]

Metabolomic profiling identifies novel associations with Electrolyte and Acid-Base Homeostatic patterns

Cristina Menni[1,*], Linsay Mccallum[2,*], Maik Pietzner[3,4], Jonas Zierer[1,5], Alisha Aman[2], Karsten Suhre[6], Robert P Mohney[7], Massimo Mangino[1], Nele Friedrich[3], Tim D Spector[1], Sandosh Padmanabhan[2]

Supplementary material

Table of contents:

Table S1. List of metabolites significantly associated with one or more electrolytes

Table S1. List of metabolites significantly associated with one or more electrolytes

			Chloride			Potassium			Bicarbonate			Sodium		
**Super**	**Sub Pathway**	**Metabolite**	**Beta**	**SE**	**P**	**Beta**	**SE**	**P**	**Beta**	**SE**	**P**	**Beta**	**SE**	**P**
**aa**	Alanine and Aspartate Metabolism	asparagine	-0.04	0.059	5.11E-01	-0.01	0.008	1.73E-01	**0.29**	**0.053**	**3.56E-08**	0.06	0.048	2.41 E-01
**aa**	Alanine and Aspartate Metabolism	aspartate	**-0.40**	**0.089**	**7.34E-06**	-0.02	0.012	7.47E-02	-0.02	0.083	7.89E-01	-0.08	0.074	2.57 E-01
**aa**	Glutamate Metabolism	glutamine	-0.08	0.064	2.19E-01	0.00	0.008	5.59E-01	**0.50**	**0.057**	**8.79E-19**	**0.26**	**0.052**	**3.92 E-07**
**aa**	Glutathione Metabolism	5-oxoproline	-0.25	0.063	9.23E-05	-0.02	0.008	3.59E-02	**0.34**	**0.057**	**2.36E-09**	0.09	0.052	7.85 E-02
**aa**	Glycine, Serine and Threonine Metabolism	glycine	0.07	0.062	2.61E-01	-0.01	0.008	2.38E-01	**0.35**	**0.056**	**3.86E-10**	**0.28**	**0.050**	**2.50 E-08**
**aa**	Glycine, Serine and Threonine Metabolism	threonine	-0.03	0.056	5.34E-01	0.00	0.007	5.67E-01	**0.28**	**0.050**	**1.84E-08**	0.00	0.045	9.32 E-01
**aa**	Leucine, Isoleucine and Valine Metabolism	3-hydroxyisobutyrate	-0.09	0.064	1.78E-01	**-0.04**	**0.008**	**6.77E-07**	-0.07	0.059	2.61E-01	-0.14	0.052	6.70 E-03

**aa**	Leucine, Isoleucine and Valine Metabolism	isovalerate	-0.34	0.087	8.34E-05	-0.01	0.012	5.10E-01	**-0.36**	**0.081**	**8.10E-06**	-0.09	0.073	1.95 E-01
**aa**	Lysine Metabolism	lysine	-0.27	0.063	2.10E-05	0.00	0.008	8.45E-01	**0.45**	**0.057**	**2.15E-15**	0.06	0.052	2.13 E-01
**aa**	Methionine, Cysteine, SAM and Taurine Metabolism	cystathionine	0.24	0.077	2.15E-03	0.03	0.010	6.91E-03	0.00	0.070	9.87E-01	**0.39**	**0.062**	**2.67 E-10**
**aa**	Methionine, Cysteine, SAM and Taurine Metabolism	cysteine	0.20	0.076	1.00E-02	-0.02	0.010	8.37E-02	**0.33**	**0.070**	**2.35E-06**	0.15	0.063	1.52 E-02
**aa**	Methionine, Cysteine, SAM and Taurine Metabolism	cystine	0.06	0.084	4.72E-01	0.02	0.011	9.55E-02	0.10	0.077	2.08E-01	**0.31**	**0.069**	**6.78 E-06**
**aa**	Methionine, Cysteine, SAM and Taurine Metabolism	methionine sulfoxide	**-0.44**	**0.097**	**6.25E-06**	-0.02	0.013	1.57E-01	-0.22	0.092	1.54E-02	-0.18	0.083	2.92 E-02
**aa**	Methionine, Cysteine, SAM	N-acetylmethionine	**0.33**	**0.066**	**7.65E-07**	-0.03	0.009	3.63E-03	-0.02	0.061	7.84E-01	0.16	0.055	4.17 E-03

	and Taurine Metabolism													
**aa**	Methionine, Cysteine, SAM and Taurine Metabolism	N-formylmethionine	**0.37**	**0.079**	**3.22E-06**	0.00	0.010	6.56E-01	0.17	0.073	1.69E-02	0.18	0.065	6.31 E-03
**aa**	Methionine, Cysteine, SAM and Taurine Metabolism	S-methylcysteine	0.00	0.062	9.91E-01	0.00	0.008	8.50E-01	**0.26**	**0.056**	**2.68E-06**	0.12	0.051	1.41 E-02
**aa**	Phenylalanine and Tyrosine Metabolism	2-hydroxyphenylacetate	0.22	0.063	3.97E-04	0.02	0.008	6.01E-03	**-0.33**	**0.057**	**9.24E-09**	0.09	0.051	7.63 E-02
**aa**	Phenylalanine and Tyrosine Metabolism	4-hydroxyphenylpyruvate	0.18	0.082	3.21E-02	0.00	0.011	6.80E-01	**0.38**	**0.075**	**4.89E-07**	0.05	0.067	4.45 E-01
**aa**	Phenylalanine and Tyrosine Metabolism	N-acetylphenylalanine	0.04	0.069	5.85E-01	0.00	0.009	6.59E-01	**-0.33**	**0.062**	**7.35E-08**	0.06	0.056	3.19 E-01
**aa**	Phenylalanine and Tyrosine Metabolism	phenylpyruvate	0.05	0.064	4.58E-01	0.00	0.008	6.71E-01	**0.25**	**0.058**	**1.50E-05**	-0.02	0.053	6.66 E-01

**aa**	Phenylalanine and Tyrosine Metabolism	tyrosine	-0.06	0.065	3.46E-01	-0.02	0.008	3.60E-03	**0.27**	**0.059**	**5.68E-06**	0.06	0.053	2.49E-01
**aa**	Polyamine Metabolism	N-acetylputrescine	-0.16	0.064	1.37E-02	0.00	0.008	9.20E-01	-0.10	0.058	9.80E-02	**-0.23**	**0.051**	**5.61E-06**
**aa**	Tryptophan Metabolism	indolelactate	0.06	0.067	3.58E-01	**0.04**	**0.009**	**9.47E-07**	-0.13	0.061	3.73E-02	0.01	0.054	8.66E-01
**aa**	Tryptophan Metabolism	N-acetyltryptophan	-0.06	0.071	3.88E-01	0.01	0.009	1.87E-01	**-0.36**	**0.065**	**3.13E-08**	0.05	0.058	3.68E-01
**aa**	Urea cycle; Arginine and Proline Metabolism	arginine	0.01	0.062	8.68E-01	0.02	0.008	4.63E-02	**0.27**	**0.056**	**1.75E-06**	0.14	0.050	5.12E-03
**aa**	Urea cycle; Arginine and Proline Metabolism	citrulline	0.09	0.066	1.70E-01	-0.02	0.008	9.29E-03	**0.43**	**0.059**	**4.31E-13**	0.12	0.054	2.38E-02
**aa**	Urea cycle; Arginine and Proline Metabolism	dimethylarginine (SDMA + ADMA)	0.08	0.065	2.44E-01	0.00	0.008	7.42E-01	**0.29**	**0.059**	**6.93E-07**	0.15	0.053	3.87E-03
**aa**	Urea cycle; Arginine and	N-acetylcitrulline	0.09	0.069	1.81E-01	0.01	0.009	4.18E-01	0.11	0.063	7.99E-02	**0.31**	**0.056**	**3.01E-08**

	Proline Metabolism													
**aa**	Urea cycle; Arginine and Proline Metabolism	ornithine	-0.04	0.063	5.15E-01	-0.03	0.008	7.54E-04	**0.35**	**0.057**	**7.13E-10**	**0.27**	**0.051**	**2.53E-07**
**aa**	Urea cycle; Arginine and Proline Metabolism	pro-hydroxy-pro	0.15	0.067	2.18E-02	0.00	0.009	8.13E-01	0.16	0.061	8.57E-03	**0.29**	**0.054**	**1.00E-07**
**aa**	Urea cycle; Arginine and Proline Metabolism	proline	0.01	0.063	8.38E-01	-0.02	0.008	6.86E-03	0.21	0.057	3.46E-04	**0.23**	**0.051**	**6.03E-06**
**ch**	Glycolysis, Gluconeogenesis, and Pyruvate Metabolism	glycerate	**-0.38**	**0.069**	**3.33E-08**	0.00	0.009	8.09E-01	0.16	0.063	1.00E-02	-0.22	0.056	7.15E-05
**c&v**	Ascorbate and Aldarate Metabolism	oxalate	**-0.42**	**0.080**	**1.37E-07**	0.00	0.010	8.46E-01	0.31	0.073	2.90E-05	-0.18	0.066	6.19E-03
**c&v**	Ascorbate and Aldarate Metabolism	threonate	**-0.43**	**0.074**	**4.79E-09**	**-0.05**	**0.010**	**2.24E-06**	0.17	0.068	1.25E-02	-0.17	0.060	4.74E-03

**c&v**	Hemoglobin and Porphyrin Metabolism	bilirubin (E,E)*	-0.10	0.062	1.04E-01	**-0.05**	**0.008**	**8.45E-10**	0.14	0.056	1.59E-02	-0.02	0.050	6.69 E-01
**c&v**	Tocopherol Metabolism	alpha-tocopherol	0.01	0.064	8.25E-01	-0.02	0.008	2.97E-02	**0.32**	**0.058**	**5.28E-08**	0.04	0.052	4.49 E-01
**e**	TCA Cycle	2-methylcitrate/homocitrate	-0.01	0.061	9.15E-01	0.01	0.008	4.14E-01	**-0.26**	**0.055**	**2.19E-06**	-0.03	0.049	5.18 E-01
**e**	TCA Cycle	cis-aconitate	-0.16	0.072	2.64E-02	**-0.05**	**0.009**	**1.59E-08**	-0.07	0.066	2.94E-01	-0.13	0.059	2.35 E-02
**e**	TCA Cycle	citrate	-0.11	0.067	1.08E-01	**-0.05**	**0.009**	**1.52E-08**	-0.07	0.061	2.38E-01	-0.17	0.054	2.07 E-03
**e**	TCA Cycle	fumarate	-0.18	0.065	6.31E-03	**-0.07**	**0.008**	**7.24E-16**	-0.09	0.060	1.28E-01	-0.02	0.053	7.20 E-01
**e**	TCA Cycle	malate	-0.14	0.069	3.67E-02	**-0.04**	**0.009**	**6.20E-06**	0.02	0.064	7.71E-01	-0.05	0.057	4.30 E-01
**l**	Carnitine Metabolism	deoxycarnitine	0.08	0.063	2.14E-01	0.02	0.008	2.32E-02	0.12	0.057	3.05E-02	**0.24**	**0.050**	**1.48 E-06**
**l**	Fatty Acid Metabolism (Acyl Carnitine)	decanoylcarnitine	-0.15	0.064	2.19E-02	**-0.04**	**0.008**	**5.12E-06**	0.18	0.058	2.33E-03	0.01	0.052	8.31 E-01
**l**	Fatty Acid Metabolism (Acyl Carnitine)	laurylcarnitine	-0.16	0.064	1.26E-02	**-0.04**	**0.008**	**2.24E-06**	0.14	0.058	1.33E-02	-0.01	0.052	7.84 E-01

**I**	Fatty Acid Metabolism (Acyl Carnitine)	linoleoylcarnitine*	-0.11	0.063	6.72E-02	**-0.05**	**0.008**	**1.29E-10**	0.11	0.057	5.32E-02	0.07	0.051	1.57 E-01
**I**	Fatty Acid Metabolism (Acyl Carnitine)	myristoleoylcarnitine*	-0.15	0.067	2.49E-02	**-0.05**	**0.009**	**3.53E-09**	0.19	0.061	1.71E-03	-0.04	0.055	5.09 E-01
**I**	Fatty Acid Metabolism (Acyl Carnitine)	octanoylcarnitine	-0.12	0.064	5.30E-02	**-0.04**	**0.008**	**2.97E-06**	0.19	0.059	1.01E-03	0.03	0.052	5.88 E-01
**I**	Fatty Acid Metabolism (Acyl Carnitine)	oleoylcarnitine	-0.15	0.070	3.38E-02	**-0.07**	**0.009**	**1.83E-13**	0.11	0.064	9.28E-02	0.00	0.057	9.53 E-01
**I**	Fatty Acid Metabolism (Acyl Carnitine)	palmitoylcarnitine	-0.18	0.071	9.51E-03	**-0.04**	**0.009**	**1.59E-06**	0.05	0.065	4.80E-01	-0.02	0.058	7.36 E-01
**I**	Fatty Acid, Branched	15-methylpalmitate	-0.05	0.063	3.89E-01	**-0.04**	**0.008**	**5.94E-06**	0.15	0.057	1.09E-02	-0.04	0.051	4.74 E-01
**I**	Fatty Acid, Branched	17-methylstearate	-0.07	0.061	2.58E-01	**-0.04**	**0.008**	**2.33E-06**	0.16	0.055	4.06E-03	-0.04	0.049	4.29 E-01
**I**	Fatty Acid, Dicarboxylate	dodecanedioate	-0.10	0.061	9.96E-02	**-0.04**	**0.008**	**5.90E-07**	0.09	0.056	1.30E-01	0.04	0.050	4.24 E-01
**I**	Fatty Acid, Dicarboxylate	hexadecanedioate	-0.18	0.069	9.57E-03	**-0.06**	**0.009**	**3.47E-12**	0.07	0.064	3.04E-01	-0.14	0.057	1.10 E-02

**I**	Fatty Acid, Dicarboxylate	octadecanedioate	-0.19	0.062	2.04E-03	**-0.04**	**0.008**	**4.38E-07**	0.01	0.057	9.03E-01	-0.08	0.050	1.14 E-01
**I**	Fatty Acid, Dicarboxylate	pimelate (heptanedioate)	-0.22	0.065	7.47E-04	0.00	0.009	8.38E-01	**-0.28**	**0.060**	**2.03E-06**	-0.11	0.054	3.48 E-02
**I**	Fatty Acid, Dicarboxylate	sebacate (decanedioate)	-0.12	0.079	1.36E-01	**-0.05**	**0.010**	**6.31E-07**	0.04	0.072	5.92E-01	0.04	0.064	5.56 E-01
**I**	Fatty Acid, Dicarboxylate	suberate (octanedioate)	**-0.29**	**0.066**	**1.49E-05**	0.00	0.009	6.60E-01	**-0.34**	**0.061**	**3.92E-08**	-0.14	0.055	1.31 E-02
**I**	Fatty Acid, Dicarboxylate	tetradecanedioate	-0.15	0.063	2.05E-02	**-0.05**	**0.008**	**1.84E-10**	0.07	0.058	2.15E-01	-0.10	0.052	5.35 E-02
**I**	Fatty Acid, Monohydroxy	16-hydroxypalmitate	-0.19	0.061	2.01E-03	**-0.05**	**0.008**	**2.23E-09**	0.11	0.056	4.60E-02	-0.12	0.050	1.92 E-02
**I**	Fatty Acid, Monohydroxy	2-hydroxydecanoate	-0.08	0.060	1.80E-01	**-0.04**	**0.008**	**8.62E-07**	0.09	0.055	9.38E-02	-0.01	0.049	8.66 E-01
**I**	Fatty Acid, Monohydroxy	3-hydroxydecanoate	-0.17	0.062	5.32E-03	**-0.06**	**0.008**	**1.55E-13**	0.11	0.057	4.53E-02	-0.10	0.051	4.07 E-02
**I**	Fatty Acid, Monohydroxy	3-hydroxylaurate	-0.19	0.063	2.97E-03	**-0.06**	**0.008**	**1.31E-12**	0.17	0.058	4.06E-03	-0.13	0.051	1.25 E-02
**I**	Fatty Acid, Monohydroxy	3-hydroxyoctanoate	-0.21	0.060	3.77E-04	**-0.04**	**0.008**	**1.24E-06**	-0.10	0.056	8.01E-02	-0.08	0.050	9.06 E-02
**I**	Fatty Acid, Monohydroxy	3-hydroxysebacate	-0.11	0.069	1.24E-01	**-0.05**	**0.009**	**4.48E-09**	-0.05	0.063	4.07E-01	-0.05	0.056	4.16 E-01
**I**	Glycerol Metabolism	glycerol	-0.05	0.065	4.41E-01	**-0.04**	**0.008**	**9.01E-08**	-0.03	0.060	6.44E-01	-0.08	0.053	1.44 E-01

I	Ketone Bodies	3-hydroxybutyrate (BHBA)	-0.08	0.064	2.20E-01	**-0.06**	**0.008**	**5.62E-13**	0.05	0.059	4.34E-01	-0.17	0.052	1.07 E-03
I	Long Chain Fatty Acid	10-heptadecenoate (17:1n7)	-0.06	0.063	3.37E-01	**-0.05**	**0.008**	**1.07E-08**	0.13	0.058	2.44E-02	-0.09	0.052	7.66 E-02
I	Long Chain Fatty Acid	10-nonadecenoate (19:1n9)	-0.03	0.061	6.11E-01	**-0.05**	**0.008**	**2.46E-09**	0.09	0.056	9.73E-02	-0.08	0.050	1.04 E-01
I	Long Chain Fatty Acid	arachidate (20:0)	-0.09	0.056	1.17E-01	**-0.03**	**0.007**	**1.28E-05**	0.10	0.051	4.32E-02	-0.04	0.045	4.31 E-01
I	Long Chain Fatty Acid	eicosenoate (20:1)	-0.10	0.060	9.06E-02	**-0.05**	**0.008**	**4.30E-11**	0.08	0.055	1.38E-01	-0.11	0.049	2.13 E-02
I	Long Chain Fatty Acid	margarate (17:0)	-0.07	0.062	2.33E-01	**-0.04**	**0.008**	**9.19E-08**	0.14	0.057	1.18E-02	-0.07	0.051	1.86 E-01
I	Long Chain Fatty Acid	myristate (14:0)	-0.08	0.062	2.23E-01	**-0.05**	**0.008**	**1.20E-09**	0.11	0.057	5.15E-02	-0.10	0.051	4.09 E-02
I	Long Chain Fatty Acid	myristoleate (14:1n5)	-0.10	0.064	1.20E-01	**-0.06**	**0.008**	**1.97E-14**	0.18	0.059	2.14E-03	-0.11	0.052	3.90 E-02
I	Long Chain Fatty Acid	nonadecanoate (19:0)	-0.10	0.059	8.56E-02	**-0.04**	**0.008**	**3.51E-06**	0.18	0.054	7.12E-04	-0.02	0.048	6.77 E-01
I	Long Chain Fatty Acid	oleate/vaccenate (18:1)	-0.06	0.062	3.60E-01	**-0.05**	**0.008**	**1.89E-11**	0.10	0.057	6.60E-02	-0.10	0.051	4.41 E-02
I	Long Chain Fatty Acid	palmitate (16:0)	-0.12	0.063	5.41E-02	**-0.04**	**0.008**	**2.80E-07**	0.12	0.058	3.70E-02	-0.12	0.052	1.66 E-02
I	Long Chain Fatty Acid	palmitoleate (16:1n7)	-0.11	0.065	8.26E-02	**-0.05**	**0.008**	**4.74E-10**	0.16	0.060	8.02E-03	-0.14	0.054	7.59 E-03

**I**	Long Chain Fatty Acid	stearate (18:0)	-0.11	0.061	7.55E-02	**-0.04**	**0.008**	**2.77E-06**	0.16	0.055	4.81E-03	-0.06	0.049	2.63E-01
**I**	Lysolipid	1-arachidonoyl-GPA (20:4)	-0.19	0.066	3.36E-03	**-0.05**	**0.009**	**9.47E-08**	0.10	0.061	8.93E-02	-0.06	0.054	3.11E-01
**I**	Lysolipid	1-docosapentaenoyl-GPC (22:5n3)*	-0.11	0.064	8.61E-02	0.02	0.008	6.18E-03	**0.34**	**0.057**	**2.43E-09**	0.14	0.051	5.49E-03
**I**	Lysolipid	1-linoleoyl-GPA (18:2)*	-0.15	0.070	3.76E-02	**-0.04**	**0.009**	**2.74E-06**	0.20	0.065	1.86E-03	0.06	0.058	2.65E-01
**I**	Lysolipid	1-palmitoyl-GPE (16:0)	-0.24	0.061	6.62E-05	0.00	0.008	9.83E-01	-0.03	0.056	5.62E-01	**-0.21**	**0.049**	**1.26E-05**
**I**	Medium Chain Fatty Acid	10-undecenoate (11:1n1)	-0.10	0.063	1.13E-01	**-0.04**	**0.008**	**5.60E-06**	0.13	0.058	2.32E-02	-0.05	0.051	3.48E-01
**I**	Medium Chain Fatty Acid	5-dodecenoate (12:1n7)	-0.14	0.062	2.00E-02	**-0.06**	**0.008**	**1.83E-13**	0.21	0.057	2.31E-04	-0.07	0.051	1.46E-01
**I**	Medium Chain Fatty Acid	caprate (10:0)	-0.10	0.059	8.62E-02	**-0.05**	**0.008**	**2.55E-09**	0.09	0.054	9.31E-02	-0.02	0.049	6.95E-01
**I**	Medium Chain Fatty Acid	caprylate (8:0)	-0.09	0.058	1.39E-01	**-0.04**	**0.007**	**2.64E-07**	0.07	0.053	1.97E-01	0.01	0.047	8.49E-01
**I**	Medium Chain Fatty Acid	laurate (12:0)	-0.09	0.058	1.08E-01	**-0.04**	**0.007**	**2.55E-09**	0.13	0.054	1.96E-02	-0.03	0.048	4.76E-01
**I**	Phospholipid Metabolism	1-palmitoyl-2-arachidonoyl-GPE (16:0/20:4)*	-0.09	0.062	1.56E-01	0.00	0.008	8.40E-01	-0.03	0.057	5.88E-01	**-0.24**	**0.050**	**1.65E-06**

**I**	Phospholipid Metabolism	1-palmitoyl-2-docosahexaenoyl-GPE (16:0/22:6)*	-0.14	0.062	2.20E-02	-0.01	0.008	4.54E-01	-0.05	0.056	3.97E-01	**-0.27**	**0.049**	**6.94 E-08**
**I**	Phospholipid Metabolism	1-palmitoyl-2-palmitoleoyl-GPC (16:0/16:1)*	-0.13	0.062	3.88E-02	0.02	0.008	4.96E-02	-0.06	0.056	3.28E-01	**-0.22**	**0.050**	**1.01 E-05**
**I**	Phospholipid Metabolism	glycerophosphoinositol*	-0.09	0.065	1.90E-01	0.01	0.008	1.03E-01	**0.26**	**0.059**	**1.04E-05**	0.02	0.053	7.24 E-01
**I**	Phospholipid Metabolism	glycerophosphorylcholine (GPC)	-0.20	0.064	2.00E-03	0.01	0.008	2.89E-01	**0.27**	**0.057**	**1.64E-06**	0.11	0.051	2.99 E-02
**I**	Polyunsaturated Fatty Acid (n3 and n6)	dihomo-linoleate (20:2n6)	-0.10	0.058	9.49E-02	**-0.04**	**0.007**	**1.23E-08**	0.08	0.053	1.16E-01	-0.08	0.048	9.03 E-02
**I**	Polyunsaturated Fatty Acid (n3 and n6)	docosadienoate (22:2n6)	-0.10	0.054	5.32E-02	**-0.04**	**0.007**	**4.57E-08**	0.06	0.049	2.53E-01	-0.08	0.044	6.27 E-02
**I**	Polyunsaturated Fatty Acid (n3 and n6)	linoleate (18:2n6)	-0.09	0.058	1.27E-01	**-0.04**	**0.007**	**2.66E-09**	0.12	0.053	2.32E-02	-0.07	0.048	1.63 E-01
**I**	Polyunsaturated Fatty Acid (n3 and n6)	linolenate [alpha or gamma; (18:3n3 or 6)]	-0.11	0.060	7.13E-02	**-0.05**	**0.008**	**1.94E-12**	0.14	0.055	1.47E-02	-0.08	0.050	9.73 E-02

**l**	Polyunsaturated Fatty Acid (n3 and n6)	stearidonate (18:4n3)	-0.18	0.067	7.00E-03	**-0.05**	**0.009**	**6.35E-09**	0.19	0.062	2.72E-03	-0.08	0.055	1.35 E-01
**l**	Sphingolipid Metabolism	sphingomyelin (d18:1/20:0, d16:1/22:0)*	0.04	0.065	4.93E-01	**0.04**	**0.008**	**1.20E-06**	-0.08	0.060	1.73E-01	0.09	0.053	1.06 E-01
**l**	Steroid	21-hydroxypregnenolone disulfate	0.16	0.066	1.33E-02	0.00	0.009	6.11E-01	0.00	0.060	9.43E-01	**0.28**	**0.053**	**2.26 E-07**
**l**	Steroid	5alpha-pregnan-3beta,20alpha-diol disulfate	0.28	0.064	1.84E-05	0.01	0.008	4.77E-01	**-0.31**	**0.058**	**1.18E-07**	0.04	0.052	4.59 E-01
**l**	Steroid	dehydroisoandrosterone sulfate (DHEA-S)	0.10	0.072	1.53E-01	0.01	0.009	1.45E-01	0.04	0.066	5.63E-01	**0.25**	**0.058**	**1.61 E-05**
**n**	Pyrimidine Metabolism, Orotate containing	dihydroorotate	-0.05	0.068	4.32E-01	**-0.05**	**0.009**	**1.56E-07**	0.14	0.062	2.74E-02	0.21	0.055	1.10 E-04
**n**	Pyrimidine Metabolism, Orotate containing	orotate	-0.11	0.068	9.00E-02	**-0.04**	**0.009**	**2.16E-07**	-0.06	0.062	3.43E-01	-0.07	0.055	1.80 E-01
**n**	Pyrimidine Metabolism, Uracil containing	5-methyluridine (ribothymidine)	0.21	0.065	1.17E-03	0.00	0.008	8.55E-01	0.05	0.059	4.27E-01	**0.25**	**0.052**	**1.55 E-06**

**p**	Dipeptide	prolylproline	-0.12	0.062	6.27E-02	0.01	0.008	8.72E-02	**-0.30**	**0.057**	**1.31E-07**	-0.10	0.051	4.22 E-02
**p**	Gamma-glutamyl aa	gamma-glutamyl-epsilon-lysine	**-0.30**	**0.067**	**9.11E-06**	0.00	0.009	8.19E-01	**0.39**	**0.061**	**1.28E-10**	0.14	0.055	1.03 E-02
**p**	Gamma-glutamyl aa	gamma-glutamylglutamine	-0.26	0.064	7.25E-05	-0.01	0.008	2.53E-01	**0.43**	**0.059**	**2.07E-13**	0.07	0.053	1.89 E-01
**p**	Gamma-glutamyl aa	gamma-glutamylmethionine	0.01	0.065	8.69E-01	-0.02	0.008	2.02E-02	**0.38**	**0.059**	**2.37E-10**	0.06	0.054	2.28 E-01
**p**	Gamma-glutamyl aa	gamma-glutamylthreonine*	-0.13	0.064	3.64E-02	0.00	0.008	6.75E-01	**0.44**	**0.058**	**2.00E-14**	0.03	0.053	5.86 E-01
**p**	Gamma-glutamyl aa	gamma-glutamyltyrosine	-0.03	0.068	6.18E-01	-0.01	0.009	2.50E-01	**0.29**	**0.062**	**3.36E-06**	0.19	0.055	4.47 E-04
**x**	Bacterial/Fungal	tartronate (hydroxymalonate)	**-0.42**	**0.077**	**5.52E-08**	0.00	0.010	9.59E-01	0.29	0.070	3.41E-05	-0.15	0.063	1.91 E-02
**x**	Chemical	4-hydroxychlorothalonil	0.00	0.071	9.76E-01	0.03	0.009	2.01E-03	**-0.30**	**0.065**	**3.20E-06**	0.10	0.058	7.22 E-02
**x**	Food Component/Plant	2-isopropylmalate	-0.11	0.064	7.52E-02	**-0.05**	**0.008**	**2.53E-08**	-0.17	0.058	3.59E-03	-0.12	0.052	1.78 E-02
**x**	Food Component/Plant	methyl indole-3-acetate	0.19	0.072	7.69E-03	0.03	0.009	2.43E-04	**-0.40**	**0.065**	**7.05E-10**	0.08	0.057	1.58 E-01
